# Isolation and characterization of a new naturally immortalized human breast carcinoma cell line, KAIMRC1

**DOI:** 10.1186/s12885-017-3812-5

**Published:** 2017-11-29

**Authors:** Rizwan Ali, Nosaibah Samman, Hajar Al Zahrani, Atef Nehdi, Sabhi Rahman, Abdul Latif Khan, Mohamed Al Balwi, Lolwah Abdullah Alriyees, Manal Alzaid, Ahmed Al Askar, Mohamed Boudjelal

**Affiliations:** 1 Medical Research Core Facility and Platforms (MRCFP), King Abdullah International Medical Research Center (KAIMRC), National Guard Health Affairs (NGHA), P.O. Box 22490, Riyadh, 11426 Saudi Arabia; 2 King Abdullah International Medical Research Center/ King Saud bin Abdulaziz University for Health Sciences (KSAU-HS), King Abdulaziz Medical City (KAMC), NGHA, Riyadh, 11426 Saudi Arabia; 3 Department of Pathology and Laboratory Medicine, King Abdullah Medical City (KAMC), NGHA, Riyadh, 11426 Saudi Arabia; 4 Department of Surgery, KAMC, NGHA, Riyadh, 11426 Saudi Arabia

**Keywords:** Breast cancer, Characterization, Isolation, Drug treatment, Gene expression, Cell line, Natural transformation, Immortalization, BRCA1, BRCA2

## Abstract

**Background:**

Breast cancer is one of the most common cancer and a leading cause of death in women. Up to date the most commonly used breast cancer cell lines are originating from Caucasians or Afro-Americans but rarely cells are being derived from other ethnic groups. Here we describe for the first time the establishment of a naturally transformed breast cancer cell line, KAIMRC1 from an Arab woman of age 62 suffering from stage IIB breast cancer (T2N1M0). Moreover, we have characterized these cells for the biological and molecular markers, induction of MAPK pathways as well as its response to different commercially available drugs and compounds.

**Methods:**

Breast cancer tissue sections were minced and cultured in media for several weeks. KAIMRC1 cells were successfully isolated from one of the primary breast tumor tissue cultures without any enzymatic digestion. To study the growth characteristics of the cells, wound healing assay, clonogenic assay, cell proliferation assays and live cell time-lapse microscopy was performed. Karyotyping, Immunophenotyping and molecular pathway specific compound treatment was also performed. A selective breast cancer gene expression panel was used to identify genes involved in the signal transduction dysregulation and malfunction of normal biological processes during breast carcinogenesis.

**Results:**

These cells are ER/PR-positive and HER2-negative. The epithelial nature of these cells was confirmed by flow cytometry analysis using epithelial cell markers. They are cuboidal in shape and relatively smaller in size as compared to established cell lines, MCF-7, MDA MB-231 and the normal breast cell line, MCF-10A. In normal cell culture conditions these cells showed the capability of growing both in monolayer as well as in 3-D conformation. They showed a doubling time in vitro of approximately 24 h. They exhibit a modal karyotype of 58–63,X with abnormalities in a couple of chromosomes. KAIMRC1 cells were found to be more responsive to drug treatment in vitro in comparison to the established MDA MB-231 and MCF-7 cell lines.

**Conclusions:**

In conclusion we have isolated and characterized a new naturally immortalized breast cell line, KAIMRC1 with a potential to play a key role in opening up novel avenues towards the understanding of breast carcinoma.

**Electronic supplementary material:**

The online version of this article (10.1186/s12885-017-3812-5) contains supplementary material, which is available to authorized users.

## Background

Breast cancer is the most common neoplasm and leading cause of death in women [1, 2]. The most common type of breast cancer is ductal carcinoma [3]. Breast cancer is by far the number one diagnosed cancer in women in the kingdom of Saudi Arabia (KSA). According to the annual cancer incident report maintained by Saudi Cancer Registry (SCR) there were 1853 female breast cancer cases accounting for 29.1% of all newly diagnosed female cancers (6364) in the year 2013. Infiltrating ductal carcinoma was 78.6% of all the breast cancer types and the median age at diagnosis was 50 years (www.chs.gov.sa/Ar/HealthCenters/NCC/CancerRegistry/CancerRegistryReports/2013.pdf).

Different experimental models are being used to study breast cancer that includes animal models, cell lines and biopsies from the tumor itself. Although they all provide great information and enabled outstanding discoveries to understand the disease and to develop powerful treatments; the hunt for safer and more potent drugs is still ongoing. In almost all of the studied models, established cell lines are generally considered to be invaluable tools. They have enabled researchers to identify and discover novel pathways leading to suppression of cancer growth and metastases. The most commonly used breast cancer cell lines were established in the last century, and only a few breast cancer cell lines have been established more recently. This lag is mainly due to the difficulties in culturing breast cancer cells without their surrounding stromal cells. The first human breast carcinoma cell line was established in 1958 [4], since then many attempts have been made to establish additional permanent breast tumor cell lines. The main advantage is that these cells are directly isolated from the tumor site and associated pathology is available. Human breast tumor cell lines, however, are difficult to establish in culture [5, 6]. One of the main reasons is the molecular heterogeneity of the cell population making up breast cancer tissue. Explanting usually gives a mixed population of fibroblasts and epithelial cells. Fibroblast cells grow faster and eventually overcome epithelial cells. In addition most of these breast tumor derived cell lines have been established from metastatic tumors [7], raising questions about their relationship to primary tumors. Thus, there is a need to establish more breast cancer cell lines that are representative of the primary tumor and which also have a similar diverse phenotype. Many research groups around the world are trying to establish new cell lines. For example, Gazdar and colleagues, developed a panel of tumor cell lines along with paired non-malignant cell lines or strains collected from breast cancers, predominantly primary tumors [8]. This included 18 cell lines derived from primary tumors and three derived from metastatic lesions and for the majority of them they established one or more corresponding non-malignant cell strains. Another study, [9] described the development of five breast cancer cell lines from a breast cancer tissue derived from a single patient. Although these were derived from a single tumor, all five breast cancer cell lines displayed different antigenic expression profiles, tumorigenicity and organ specific metastatic abilities.

Until now the most widely used breast cancer cell lines were originally from Caucasians or African Americans such as MCF-7, ZR-75-30, T47D and MDA MB-231 but rarely cell lines are derived from other ethnic group. However even if they have been established, they are not widely used by researchers. NIPBC-1 and NIPBC-2, triple negative breast cancer cell lines were established from primary tumors of two young breast cancer patients aged 39 and 38 years respectively, diagnosed for infiltrating duct carcinoma of the breast [10]. These cell lines showed luminal origin with expression of epithelial specific antigen and cytokeratin 18 and the presence of microfilaments and secretary vesicles, microvilli, tight junctions and desmosomes. Anchorage independent growth, invasion of matrigel coated membranes, presence of CD 24^−^/44^+^ breast cancer stem cells and capability of producing mammosphere in-vitro was identified in both the cell lines. On the other hand, three Chinese breast cancer cell lines, namely, BC-019, BC-020 and BC-021 have also been established and characterized. They were established from breast invasive ductal carcinoma tissues. They grow as adherent monolayer with characteristic epithelial morphology and are ER^−^, PR^−^ and Her-2^+^ with high hyperdiploidy and complex chromosomal rearrangements, and displayed aggressive tumorigencity [11].

In most of the breast cancer cell line establishment studies the emphasis is given only on the characterization at the molecular and chromosomal level, and their tumorigenic properties in vitro. To our knowledge, less attention has been given to the study of drug response and the molecular pathways associated with these responses. In this current work we put our focus on study in the drug response of KAIMRC1 cells targeting a specific pathway.

With the advent of personalized medicine, it is of great importance that representative cell lines of different ethnic groups are used to better understand and characterize difficult to treat cancers, in particular breast cancer. Keeping this in mind it is necessary to establish new cell lines from ethnic groups other than Caucasians and Afro-Americans.

Here we describe in our study for the first time the establishment of a naturally transformed breast cancer cell line from an Arab woman of 62 years diagnosed with ductal breast carcinoma. This novel cell line termed KAIMRC1, was characterized for biological and molecular markers, induction of MAPK pathways as well as its response to different commercially available drugs and compounds.

## Methods

### Clinical history of the patient

A 63-year-old female was diagnosed with right breast central mass infiltrating ductal carcinoma. Right simple mastectomy was performed with SBR grade 2/3 (architectural score 2, nuclear grade 2, mitotic score 2). Ductal carcinoma in situ (DCIS) was present (intermediate to high nuclear grade micropapillary, cribriform and comedo pattern); 25%, invasive carcinoma size was 4 cm in maximum dimension, lymphovascular invasion was not present, microcalcification was present and pathological stage was pT2 pNa pMx. Sentinel lymph node biopsy showed 1 out of 3 sentinel lymph nodes positive for metastatic carcinoma. Axillary lymph node dissection of 12 lymph nodes was negative for metastasis. Immunohistochemistry analysis revealed ER (SP1) positivity with strong staining in almost all cells; score 3+, Pr (IE2) positivity with weak to moderate staining in approximately 50% of cells; score 2+, Her2 neu (4B5) negativity with incomplete, barely perceptible membrane staining in <10% cells; score 0 and Ki-67 (MIB1) staining with proliferation index of approximately 10%. The final pathological stage according to the 7th AJCC TNM staging system: pT2 pN1a M0 (Stage 2B). The oncotype DX showed recurrence score of 16 which is considered low. The patient didn’t receive any chemotherapy but was maintained on letrozole. She is currently in remission and free of any metastases.

### Isolation and culture of breast tumor-derived cells

**Fig. 1:**
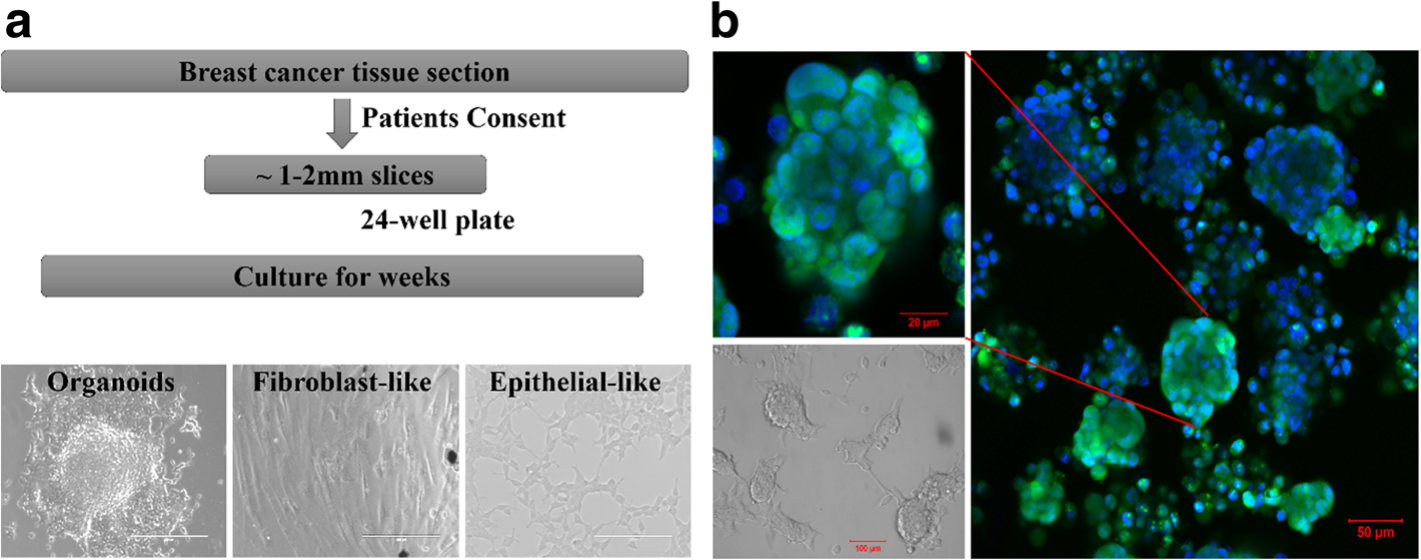
**a** Workflow of isolation of primary breast cancer cells. Breast cancer tissue sections were collected after consenting patient and cut into small 1-2 mm slices. These slices were cultured in supplemented advanced DMEM for several weeks until small organoids were visible in the vicinity of the slices. Outgrowth of fibroblast- and epithelial-like cells was visible from the organoids within 2–3 weeks of culture *(Scale bar = 200* μm*).* **b** Primary tumor epithelial cells (KAIMRC1) making dome like colonies in-vitro. Nucleus is stained with HOECHST (Blue) and Cytoplasm is stained with cell tracker (Green). In normal cell culture conditions these cells showed capability of growing both in monolayer as well as in 3-D conformation

Breast tumor-derived cells were isolated by right simple mastectomy. Informed written consent was obtained from the patient according to the Institutional Review Board (IRB) of King Abdullah International Medical Research Center (KAIMRC), Riyadh Saudi Arabia. A small tissue sample was minced into approx. 1–2 mm^3^ pieces, washed in PBS and incubated in 24-well cell culture plates in advanced Dulbecco’s Modified Eagle Medium (DMEM) supplemented with 10% Fetal Bovine Serum (FBS), 50 units/ml penicillin and 50 μg/ml streptomycin (Gibco), 2 mM L -glutamine (Gibco) at 37 °C in a humidified 5% CO_2_ atmosphere. Continuous culture of one of the tissue pieces resulted in adhesion to the culture-ware and an outgrowth of cells attached to the tissue and the plate was observed (Fig. 1). Two types of cells, epithelial like cuboidal cells and fibroblast like elongated cells were visible for the first few weeks. Later epithelial like cells started to make colonies in dome like shapes. These cell domes were isolated, picked and transferred to cell culture treated flasks. These cells were regularly passaged and expanded in the above indicated media for characterization.

### Cell lines culture

All cell lines were purchased from ATCC, USA. Human breast cancer epithelial cell lines, MDA MB-231 (HTB26) and MCF-7 (HTB-22) were maintained in supplemented advanced DMEM as described above. Human non-tumorigenic breast epithelial cell line, MCF-10A (RL-10317) was maintained in DMEM/F12 media supplemented with 5% horse serum, 0.5 μg/ml hydrocortisone, 100 ng/ml cholera toxin, 10 μg/ml insulin, 20 ng/ml EGF, 50 units/ml penicillin and 50 μg/ml streptomycin, 2 mM L-glutamine. Cells were cultured at 37 °C in a humidified 5% CO2 atmosphere.

### Growth curve

KAIMRC1, MCF-7, MDA MB-231 and MCF-10A cells were seeded at 3000 cells per well in a 48-well plate in the media described above. Media was replaced every fourth day. Cells were counted in triplicates using Vi –Cell XR (Beckman Coulter) for 4 weeks and the average counts were plotted on the growth curve.

### Soft agar and methyl cellulose colony formation assay

In case of soft agar assay, growth was examined by plating 20,000 cells on 6-well plates (Corning) containing a 0.35% agar base. The agar (Thermo Scientifc, USA) was prepared in complete DMEM, and cells were fed twice a week by adding 1.0 ml of 0.35% agar. In case of methyl cellulose assay, 5000 cells were plated in a 24-well plate containing a 0.5% methyl cellulose. Colony formation was monitored microscopically and transmitted light images were acquired thrice a week.

### Western blotting and human phospho MAP kinase profiler array

Prior to protein extraction cells were pre- incubated with 10% serum DMEM and serum-free DMEM for overnight. Western blotting analysis was performed with antibodies against Estrogen Receptor (Clone SP1; Fisher Scientific), Progesterone Receptor, Her2/ErbB2, AKT, p-AKT, p38, p-p38, ERK1/2, p-ERK1/2. All the antibodies were purchased from Cell Signaling. Signals were detected using a ChemiDoc MP System (Bio-Rad) and analyzed on ImageLab software. Sample loading was examined by probing the same membrane with anti β-actin antibody (cell Signaling). To validate results, Proteome Profiler Human Phospho-MAP Kinase Array kit (R&D Systems, Minneapolis, MN, USA) was also utilized by following the manufacturer’s instructions.

### Compound treatment

To determine the dose response effect Doxorubicin hydrochloride (Cat. No. 2252), Letrozole (Cat. No. 4382), API-2 (Cat. No. 2151), 10-DEBC hydrochloride (Cat. No. 2558), and LY294002 hydrochloride (Cat. No. 1130) were used. All compounds were purchased from Tocris, USA. Compounds were serially diluted two folds in DMSO per well in an opaque-walled 96-well plate (Costar). Three thousand cells/well in 100 μl were seeded in phenol red free DMEM (with or without FBS). Cells were incubated at 37 °C for 1–2 days with no further changes.

### Cell proliferation assay

To determine the effect of the compounds on cell proliferation, the CellTiter-Glo assay (Promega) was used according to the manufacturer’s recommendations. Luminescence was measured using the Envision plate reader (Perkin Elmer). Luminescence readings were normalized to averaged DMSO controls and expressed as a relative percentage. Data was analyzed with Graphpad Prism software and the half maximal inhibitory concentration (IC_50_) was determined. Error bars denote standard deviation (SD).

### RNA isolation and cDNA reverse transcription

Total RNA isolation from cells (exposed and non-exposed to drugs) was performed using Ambion RNA Isolation Kit (Ambion) following manufacturer instructions and RNA quantified with Nanodrop 8000 Spectrophotometer (Thermo Scientific). One microgram of total RNA was reverse transcribed into cDNA using High Capacity cDNA Reverse Transcription Kit (Applied Biosystemns) and was diluted 1:2 in ddH_2_O.

### Real time qPCR and Qiagen breast cancer panel

Real-time qPCR reactions of the target and endogenous gene were performed in triplicates for each diluted cDNA sample by using TaqMan Gene expression Master Mix (Applied Biosystems). Predesigned TaqMan Gene expression assays (Applied Biosystemns) were used and 25 μl of the reaction mix was loaded in each well of a MicroAmp Optical 96-well Reaction Plate. The plate was sealed and PCR amplification was performed on a 7900HT Fast Real time PCR System (Applied Biosystems). In case of Breast cancer gene panel (Qiagen), manufacturer’s protocol was followed. Relative changes in mRNA expression levels were calculated by the ΔΔCt method using Sequence Detection System 2.1 software (Applied Biosystems). Data was analyzed in RQ Manager 1.2.1 software (Applied Biosystems) and Microsoft Excel.

### Immunocytochemistry (ICC)

Cells were seeded 24 h prior to the experiment in μ-slide 8-well ibitreat chamber slides (Cat. 80,826, Ibidi, Germany). Cells were fixed, permeabilized and incubated with the diluted antibodies against ER, PR and HER2 overnight at 4 °C. All antibodies were purchased from Cell Signaling. Secondary FITC antibody (Life technologies) was used to detect antibody fluorescence and counter staining was performed with HOECHST (Life Technologies) to detect nucleus. Slides were imaged using LSM 780 (Zeiss, Germany).

### Confocal Laser Scanning Microscopy (cLSM)

cLSM of stained cells was performed using a Zeiss LSM 780 (Zeiss, Germany) instrument equipped with argon and In-tune lasers. Cell Tracker™ Green (Life Technologies) and FITC (Life Technologies) were detected using Argon laser at 488 nm/520–530 (ex/em) and HOECHST 33342 (Thermo Fisher Scientific) was detected using UV laser at 350 nm/460 nm (ex/em).

### Flow cytometry analysis of cell markers

Flow cytometry was performed using the FACS Canto II cytometer (BD) and subsequent analysis was performed using the FACS Diva software (BD). Briefly, 500,000 cells re-suspended in PBS were pelleted. Fluorescently labelled primary antibodies α-smooth muscle 1A4 (Abcam), CD24, CD234 (E-Cadherin), CD44, CD227, EpCAM (EBA-1), HER2, CD49c, CD116, SSEA-1, CD45, CD47, ALDH1 (LSBio), cytokeratin 18 (CK18) and SSEA-1 (CD15) were purchased from BD Pharmingen unless otherwise specified. Cells were incubated with antibodies and analyzed on the flow cytometer. For the flow cytometry analysis, cells were gated based on FSC and SSC properties and Interval gates on histogram plots were set-up for positive cell populations in FITC, PE and APC-Cy7 channels.

### Scanning Electron Microscopy (SEM)

Cells were grown and fixed as described above. Cells were then dehydrated with graded concentrations of Ethanol (Sigma) and transferred to appropriate carbon taped stubs (Ted Pella, USA) for SEM. To enhance the electron conductivity, samples were coated with gold/palladium (Au/Pd) by sputter coating and examined on a FEI NanoSEM 450 scanning electron microscope at 10 kV.

## Results

### Characterization of KAIMRC1 cells

**Fig. 2:**
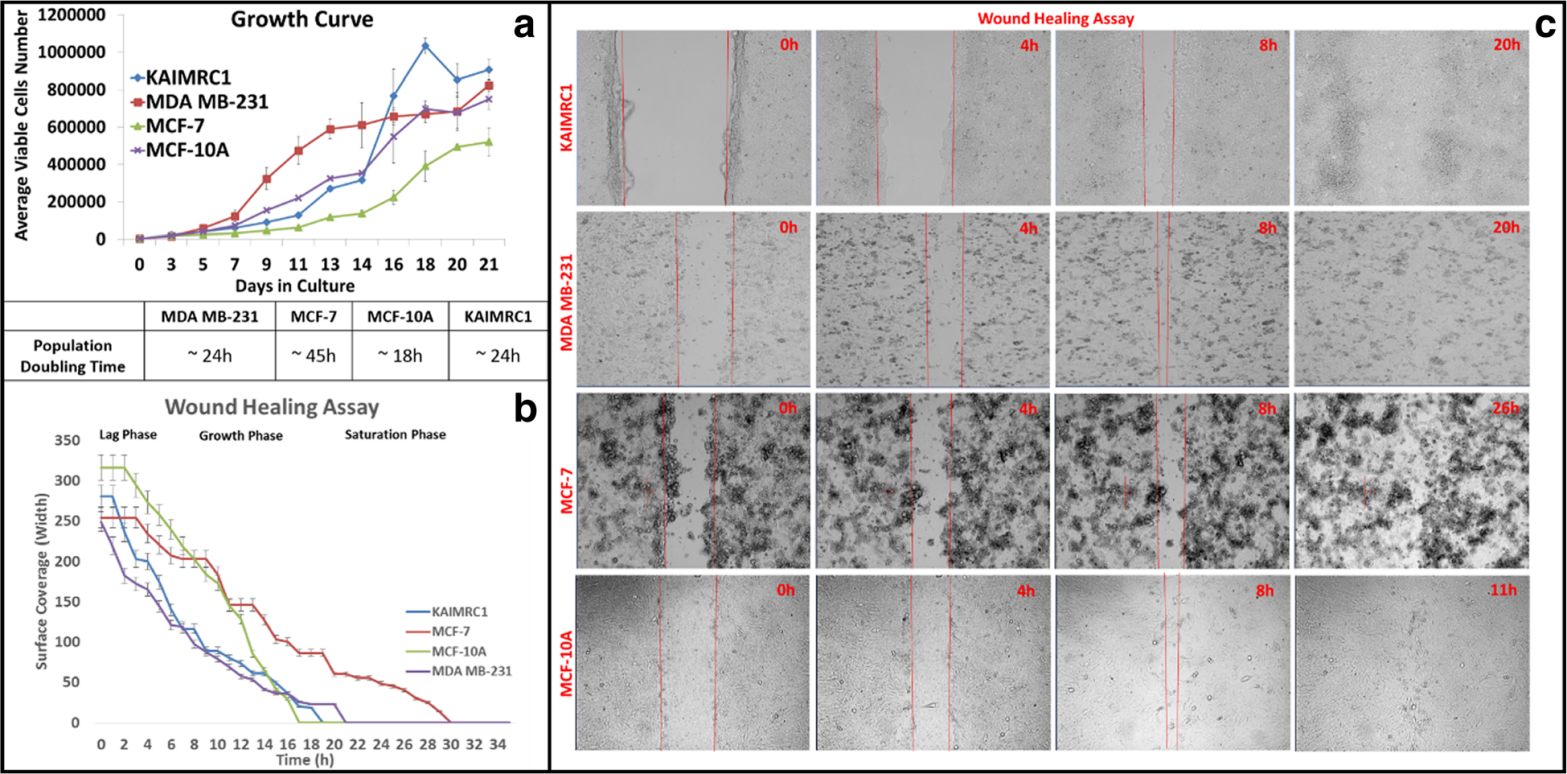
**a** Growth Characteristics of KAIMRC1 cells. The growth kinetics of KAIMRC1 cells at early passages were studied in comparison with other established breast cancer cell lines, MCF-7, MDA MB-231 and normal breast cell line MCF-10A. The average growth curves of the cell lines are shown. All the cells were seeded at 3000 cells per well in a 48-Well plate and counted in triplicates for 24 days. The population-doubling time of MDA MB-231, MCF-7, MCF-10A and KAIMRC1 cells were approximately 45, 24, 18 and 24 h, respectively. **b** The wound healing assay of KAIMRC1 cells was performed on all the four cell lines. The graph reveals that the proliferation and wound healing ability of KAIMRC1 cells was comparable to other breast cancer cell lines, MCF-7 and MDA MB-231. **c** Time-lapse images of the wound healing assay performed on the cells. A scratch was made on the confluent layer of the cells (area between the two parallel red lines) and transmitted light time-lapse imaging was performed for 2 days. Images show cells after 0 h, 4 h, 8 h and 20 h. *Scale bar = 200 μm*

Breast cancer tissue sections were obtained following consent from several patients diagnosed with breast carcinoma. Samples were cut into small 1–2 mm slices and cultured in supplemented advanced DMEM for several weeks until small organoids were visible in the vicinity of the slices. Outgrowth of fibroblast- and epithelial-like cells were visible from the organoids within 4–6 weeks of culture. Primary tumor cells showing epithelial-like morphology were successfully isolated and propagated out from one of the primary breast tumor tissue cultures. These cells were obtained without any enzymatic digestion of breast tumor tissue. They displayed cuboidal-like morphology (Fig. 1a), made dome like colonies in-vitro (Fig. 1b), and were relatively smaller in size compared to the established cell lines, MCF-7, MDA MB-231 and the normal breast cell line, MCF-10A (Fig. 2s-v). In normal cell culture conditions these cells showed capability of growing both as monolayers and as 3-D cultures. These cells were later termed KAIMRC1 cells.

### Growth characteristics

**Fig. 3:**
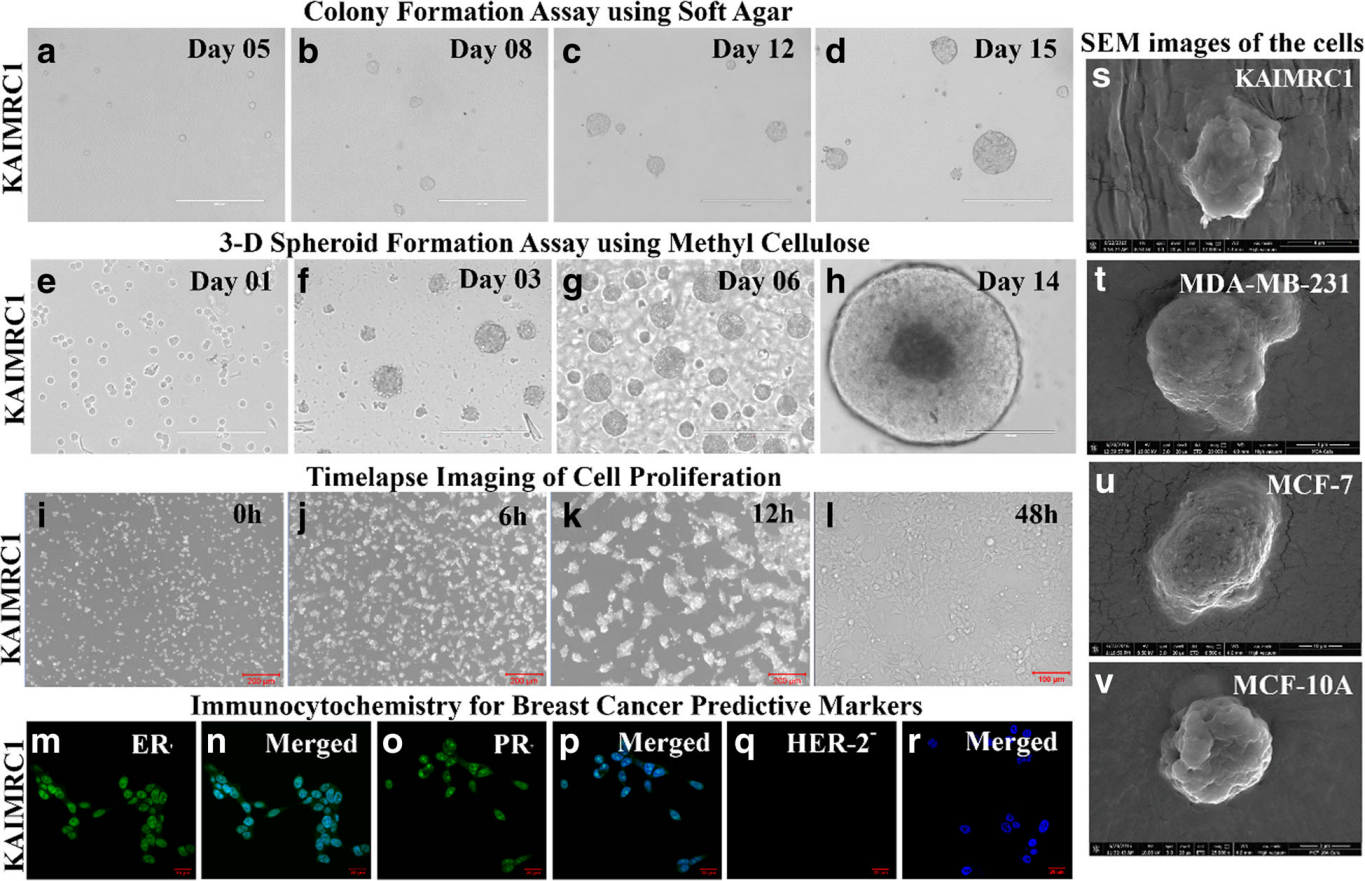
Assays to monitor cell growth and proliferation of KAIMRC1 cells. **a-d** Colony formation assay using soft agar. *Scale bar = 400 μm* ***.*** **e-h** 3-D spheroid formation assay using methyl cellulose. *Scale bar = 400 μm* ***.*** **i-l** Time-lapse imaging of cell proliferation. *Scale bar = 200 μm* ***.*** **m-r** Immunocytochemistry of breast cancer predictive markers. *Scale bar = 20 μm* ***.*** **s-v** Scanning electron microscopy (SEM) images of breast cancer cell lines, MCF-7, MDA MB-231 and normal breast cell line MCF-10A in comparison to KAIMRC1 cells

The growth kinetics of KAIMRC1 cells at passages 10–15 were studied in comparison with the other established breast cancer cell lines, MCF-7, MDA MB-231 and the normal breast cell line MCF-10A. The growth curves of these cell lines are shown in Fig. 3a. The population-doubling time of MDA MB-231, MCF-7, MCF-10A and KAIMRC1 cells were approximately 24, 45, 18 and 24 h, respectively. KAIMRC1 cells showed rapid growth similar to MCF-10A and MDA MB-231. KAIMRC1 cells grew slowly when grown sparsely and when these cells were grown in close contact with one another grew rapidly and formed domes.

### Wound-healing assay

The migration potential of KAIMRC1 cells was assessed by the wound healing assay (Fig. 3b & c) and compared with other breast cancer cell lines, MCF-7, MDA MB-231 and the normal breast cell line MCF-10A. Migration of cells and closure of wound was observed over time using time-lapse microscopy. Migration behavior of KAIMRC1 cells was found to be the fastest among the four cell lines tested. Following wound formation, KAIMRC1 cells were able to completely close the wound within 20 h.

### Clonogenic assays using methyl cellulose and soft agar

The ability of cells to make colonies in an anchorage independent manner was assessed between passages 10–15 (Fig. 2e-h). We used both soft agar and methyl cellulose methods to assess the clonogenicity of KAIMRC1 cells with success. Colonies grew faster and bigger in the methyl cellulose assay. Small colonies of KAIMRC1 cells were visible within 6 days of single cell culture in methyl cellulose and a significant increase in the number of large colonies was observed after 10–12 days of culture. Significantly KAIMRC1 cells showed ball shaped colonies for 10–15 days after which colony fusion and disintegration was evident due to extra-large sized colonies (Fig. 2d).

### Live cell time-lapse microscopy

Cells were seeded at a very low density and subjected to live cell time-lapse microscopy to observe cell proliferation behavior of KAIMRC1 cells in comparison to MCF-7, MDA MB-231 and MCF-10A. KAIMRC1 cells started to make domes by aggregating within 6 h of incubation. Interestingly, these 3-D cell domes increased in size indicating cell division for several hours before disintegration into a monolayer (Fig. 2i-l). This unique behavior of these cells is very interesting and needs further research to identify the molecular mechanisms behind it. It has also been observed that these cells do not stick firmly to plastic and glass surfaces as is common with other cell lines and can be detached easily unlike other breast cancer cell lines. These cells do not require enzymatic detachment from cell culture plates and flasks. This unique feature is very useful in order to preserve their signaling cascade and extracellular matrix (ECM) which may get effected with continuous enzymatic digestion during regular passaging [12].

### Chromosome analysis

**Fig. 4:**
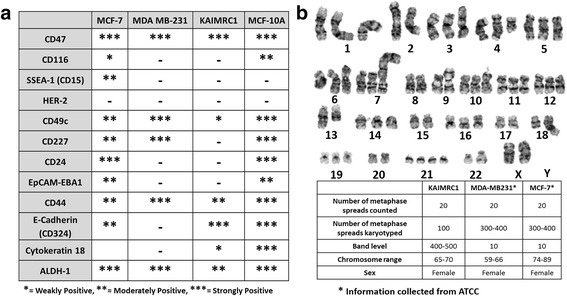
**a** Characterization of KAIMRC1 cells by flow cytometry. A panel of biomarkers were used to characterize the cells. **b** Representative Karyogram of KAIMRC1 cells. Patient karyotyping revealed presence of a composite complex karyotype including; trisomy of chromosomes X,2,3,5,6,7,8,9,10,11,12,14,15,16,17,18,19,20,21 and 22; tetrasomy of chromosomes X,2,5,6,12,17,20,21,and 22; pentasomy of chromosomes 21, and 22; additional chromosomal material on chromosomes X(p22.3),1(q44)(×2), 8(p21),11(p15); deletion of chromosome 3(q21); and additional two marker chromosomes cannot be identified. These numerical and structural chromosomal abnormalities with multi-clonality suggests existence of heterogeneity of genomic instability in this patient

KAIMRC1 cells karyotyping revealed the presence of a composite complex karyotype including; trisomy of chromosomes X, 2, 3, 5, 6, 7, 8, 9, 10, 11, 12, 14, 15, 16, 17, 18, 19, 20, 21 and 22; tetrasomy of chromosomes X, 2, 5, 6, 12, 17, 20, 21 and 22; pentasomy of chromosomes 21, and 22; additional chromosomal material on chromosomes X(p22.3), 1(q44)(×2), 8(p21), 11(p15); deletion of chromosome 3(q21); and additional two marker chromosomes cannot be identified (Fig. 4). These numerical and structural chromosomal abnormalities with multi-clonality suggests existence of heterogeneity of genomic instability in this new naturally immortalized cell line.

### Immunophenotyping

To study the phenotype of KAIMRC1 cells, we analyzed the expression of a broad panel of markers that have been shown previously to be differentially expressed in either epithelial, stem and/or cancer cells. In parallel, the same panel of markers was also run on the MCF-7 and MDA-MB-231 cell lines given that these cell lines represent in vitro cellular models of breast adenocarcinoma cells. As a control for comparative purposes, the same panel of markers was also run on MCF-10A cells; a cell line derived from a non-tumorigenic mammary gland. As Fig. 4a shows, KAIMRC1 cells were highly positive for CD47, CD324 (E-cadherin), moderately positive for CD44 suggesting that these cells display an epithelial phenotype. In addition, these cells were also positive for the cancer stem cell markers, Aldehyde Dehydrogenase 1 (ALDH1A1) [13] and CD44 [14] suggesting that these cells display some characteristics of stem-ness. KAIMRC1 cells were also found to be weakly positive for the intermediate filament protein cytokeratin 18 (CK18) which suggests that these cells are of basal type. MCF-7 were strongly positive for CD47, CD24 and ALDH1A1 and moderately positive for Site-specific Embryonic Antigen −1 (CD15), CD49c, CD227, EpCAM-EBA1, CD44 and E-Cadherin (CD324). MDA MB-231 cells were strongly positive for CD47, CD49c, CD227, CD44 and ALDH1A1. Collectively, the analysis of expression markers suggests that KAIMRC1 cells are sufficiently different from the control cell line MCF-10A and the breast adenocarcinoma cell lines, MCF-7 and MDA MB-231.

To classify KAIMRC1 cells, expression of estrogen receptor (ER), progesterone receptor (PR) and human epidermal growth factor receptor 2 (HER2) was also studied using immunocytochemistry. The cells were found to be ER/PR-positive and HER2-negative (Fig. 2m-r). This result was validated using western blot analysis (Additional file 1: Figure S1). The same result was obtained by Immuno-histochemistry of the patient sample.

### Molecular and pathway characterization of KAIMRC1 cells

We carried out several cellular assays including phopho-protein profiling, drug sensitivity and gene expression to gain deep insights into the possible pathways that may be involved and responsible for the natural transformation of KAIMRC1 cells.

### Phospho-protein profiling

**Fig. 5:**
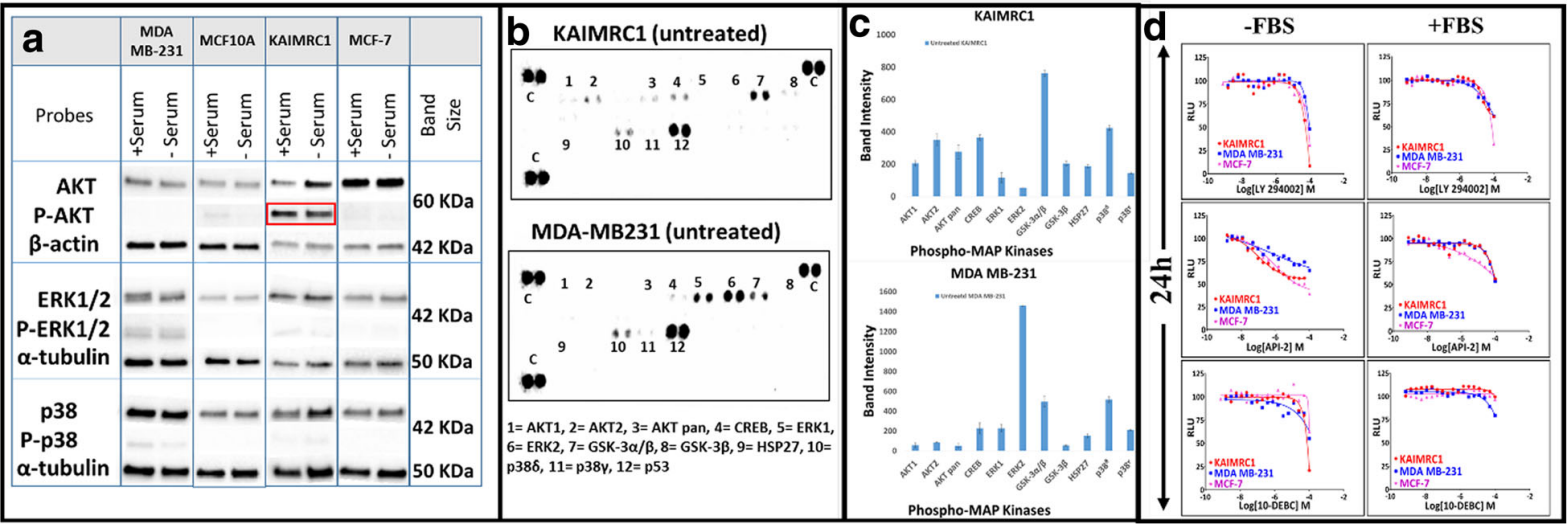
**a** Characterization of KAIMRC1 cells on protein level. Western blot analysis of breast cancer cell lines, MCF-7, MDA MB-231 and normal breast cell line MCF-10A against AKT, p-AKT, ERK, p-ERK, p38 and p-p38. Interestingly, AKT was found to be constitutively active with or without starvation. **b** Human Phospho-Mitogen-activated Protein Kinase (MAPK) Antibody Array analysis of KAIMRC1 and MDA MB-231 cells. Phosphorylation of AKT was confirmed in KAIMRC1 cells as well as strong phosphorylation of GSK-3α/β was also observed whereas, MDA MB-231 cells showed active phosphorylation of ERK, GSK-3α/β and p38δ. **c** Bar graph representation of Human Phospho-MAPK antibody array. X-axis represents selected phospho-MAP Kinases and y-axis represents intensities of the dots visible in (**b**). All experiments were done in triplicates. **d** Effect of AKT/PI3K Pathway Inhibitors on KAIMRC1 Cells. Selective inhibitor of AKT signaling, API-2; Selective inhibitor of AKT/PKB pathway, 10-DEBC Hydrochloride and PI 3 Kinase inhibitor, LY294002 were used to inhibit AKT/PI3K pathway to study the constitutively active state of AKT in KAIMRC1 cells in comparison to MDA MB-231 and MCF-7 cell lines. CellTiter-Glo® assay was performed to assess cell viability after compound treatment. The cells were treated with compounds in complete media with and without serum 24 h prior to the assay. All the three cell lines showed no response to compound treatment suggesting that AKT pathway is not the only pathway involved in the carcinogenesis of KAIMRC1 cells. X-axis = Relative light unit (RLU) and y-axis = Logarithm of the drug molarity (Log[drug]M)

Activation of oncogenic signaling proteins AKT, p38 and Extracellular signal-regulated protein kinases 1 and 2 (ERK1/2) was investigated by western blot analysis (Fig. 5a). Interestingly, in KAIMRC1 cells, AKT was found to be constitutively active with or without serum when its activity was measured using phospho-active AKT antibody. Whereas p38 and ERK1/2 showed activity in MDA MB-231 cells but not in KAIMRC1 cells. AKT is a key regulator of protein translation, transcription, cell proliferation, and apoptosis [15]. These results led us to believe that the newly isolated cancerogenic KAIMRC1 cells possess some unique mutations in comparison to other established breast cancer cell lines and that these mutations may be linked to the PI3K/AKT pathway.

To validate the western blot results we used Human Phospho-Mitogen-activated Protein Kinase (MAPK) Antibody Array on KAIMRC1 and MDA MB-231 cells (Fig. 5b and c). This array simultaneously detects the relative phosphorylation of several kinases without performing independent western blots. Active phosphorylation of AKT was confirmed in KAIMRC1 cells as well as strong phosphorylation of Glycogen synthase kinase 3α/β (GSK-3α/β). In contrast, MDA MB-231 cells showed strong phosphorylation of ERK, GSK-3α/β and p38δ. High phosphorylation levels of GSK-3α/β indicates lower activity of this kinase in the tumor cells [15]. These results suggest that the AKT/GSK3 pathway is of key importance in the carcinogenicity of KAIMRC1 cells.

### Drug sensitivity

In order to find the link between the cancerogenicity of KAIMRC1 cells and its constitutively active AKT state, we profiled a few inhibitors of the AKT and its associated pathways. LY294002; PI3-kinase inhibitor, API-2; selective inhibitor of AKT (protein kinase B; PKB), GW583340; potent dual EGFR/ErbB2 inhibitor and 10-DEBC hydrochloride; selective AKT/PKB inhibitor were used to inhibit AKT-associated pathways. The Titer-Glo® cell proliferation assay was performed to study growth inhibition of KAIMRC1 cells with and without serum for 24 h. The IC_50_ curves in Fig. 5d unfortunately showed no growth inhibition after treatment with these compounds suggesting that the AKT pathway may not be the only source of transformation of KAIMRC1 cells.

### Human breast cancer genes expression

**Fig. 6:**
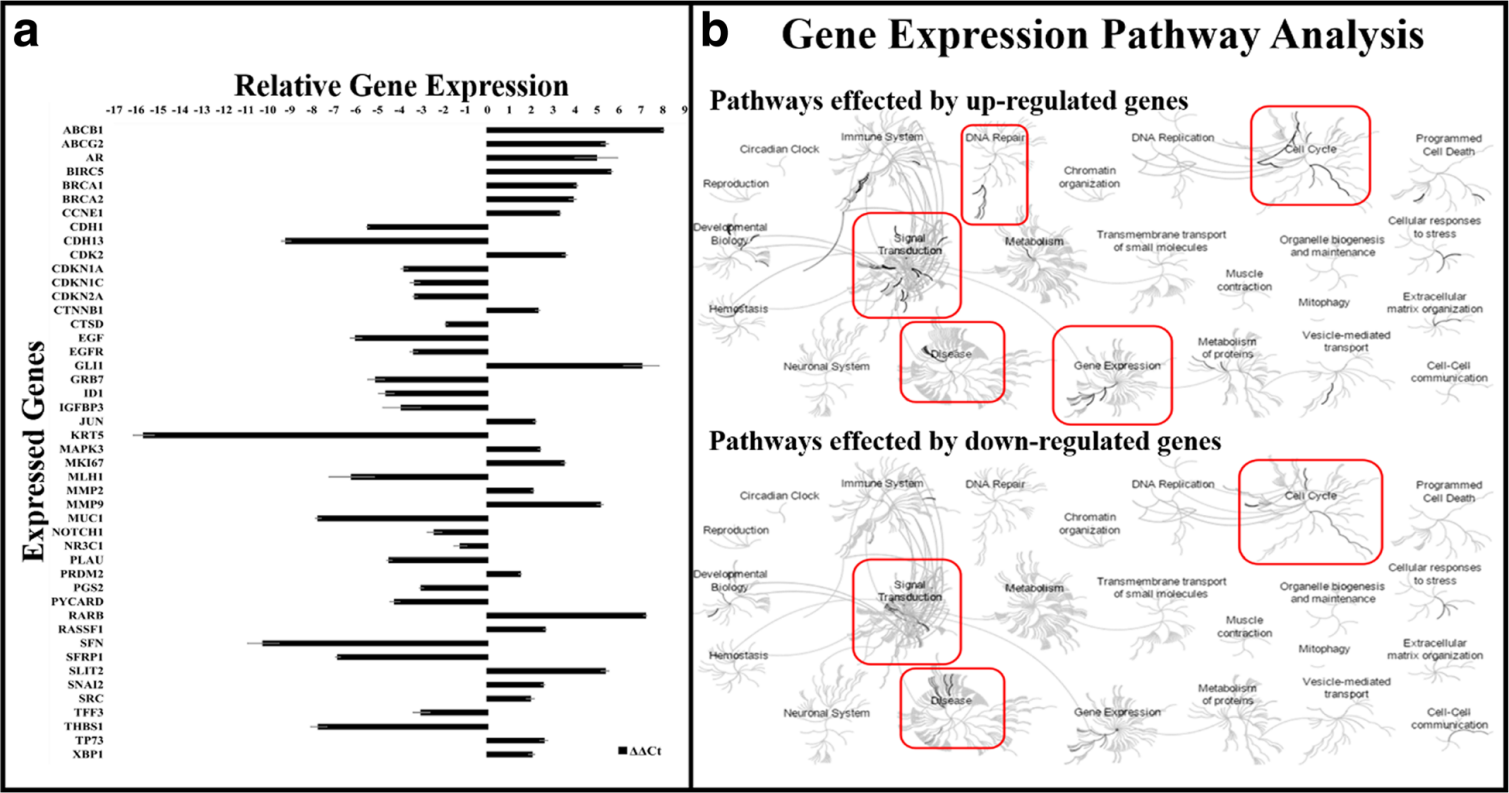
**a** Gene expression analysis of breast cancer KAIMRC1 cells utilizing breast cancer gene panel. 84 genes were analyzed by qPCR for gene expression changes out of which 46 genes showed significant changes. Data is plotted as relative up-regulation or down-regulation over normal breast MCF10A cells. Each column represents a single gene and represents data from duplicates. X-axis = Genes and y-axis = Relative gene expression (ΔΔC_t_) to normal breast cells. **b** Gene expression analysis of KAIMRC1 cells using bioinformatics pathway browser tool (Reactome). We segregated the identified genes into up- and down-regulated genes. Web-based freely available pathway analysis tool, Reactome Pathway database (reactome.org) was used to identify the pathways affected by these genes. In KAIMRC1 cells, upregulated genes were found to be activating DNA repair, signal transduction, metabolism of proteins and cell cycle related pathways whereas downregulated genes were mainly involved in signal transduction, cell cycle and disease related pathways

We used the Human Breast Cancer RT^2^ Profiler PCR Array (Applied Biosystems®) which profiles the expression of 84 key genes commonly known to be involved in the signal transduction dysregulation and malfunction of normal biological processes during breast carcinogenesis. This array contained a list of the pathway-focused genes as well as five housekeeping genes. Out of 84 genes, 46 genes showed significant gene expression changes in KAIMRC1 cells relative to normal breast MCF-10A cells (Fig. 6a). We segregated these genes into up- and down-regulated genes. Web-based freely available pathway analysis tool, Reactome Pathway database (reactome.org) was used to identify the molecular pathways affected by these genes. In KAIMRC1 cells, several upregulated genes were found to be activating DNA repair, signal transduction, metabolism of proteins and cell cycle related pathways whereas downregulated genes were mainly involved in the signal transduction, cell cycle and disease related pathways (Fig. 6b).

## Discussion

The current knowledge base around breast carcinoma progression is based on the studies performed in vivo and in vitro using available cell lines [16]. As breast cancer is a very complex and heterogeneous disease, choosing the right cell line is very important. Although the pathways leading to tumorigenesis are linked together, almost all of the available tumor-derived cell lines show heterogeneity in the acquired cellular signaling pathway or multiple pathways to immortality.

In this study we have established a naturally transformed human breast carcinoma cell line, KAIMRC1 obtained from a female patient of Saudi Arabian origin. This cell line has been isolated without any enzymatic digestion of primary breast cancer tissue. Enzymatic digestion may affect the signaling cascade and the progression of cell cycle [12, 17]. Usually the digestion affect the adhesion molecules that are critical for the cell cytoskeleton of normal as well as cancer cells. In addition it has been reported that the proteolytic degradation of extracellular matrix networks may have adverse effects on distinct signaling pathways in breast tumor tissue [18]. Thus, we assume that the isolated cell line characterized in this study has all the signaling pathways intact as no enzymatic digestion was involved in the isolation process.

Anchorage-independent growth determines the transformation capability of the cells and is the hallmark of cell transformation. KAIMRC1 cells formed colonies in both soft agar and methyl cellulose assay indicating that they have acquired a transformed phenotype. This migration ability of cells in vitro is believed to be related to several cellular in vivo behaviors, for example migration of cells during differentiation and the deviated metastatic activities of tumor cells [19].

Surgical sections obtained after breast surgery are heterogeneous and contaminated with normal and cancer associated fibroblasts (CAFs). In order to make sure that the isolated cells are of epithelial origin and cancerous, we used a panel of cell surface markers to characterize the cell line. KAIMRC1 cells were found to be positive for epithelial cell markers CD47 [20], CD324 [21] and CD44 [22]. In breast cancer, putative cancer stem cells (CSCs) with CD44 positive phenotype constitutes 12–35% of the tumor cells [23]. CD324 [21], CD44 [14, 24, 25] along with ALDH1A1 [24, 26] are believed to be CSC markers and all three were positive in KAIMRC1 cells. KAIMRC1 cells were also strongly positive for CD47 which suggests that these cells might have tumor invasion and metastasis capability [27] Enhanced expression of CD47, a transmembrane glycoprotein mediating a ‘don’t eat me’ signal, has been reported in various cancers [28–30]. It has been reported that increased expression of CD47 enables cancer cells to evade phagocytosis by macrophages and promotes the CSC phenotype [31]. Therefore, targeting CD47 has promising therapeutic potential and KAIMRC1 cell line may be used as a model to study CD47 mediated tumor invasion. Recently, researchers have used antibodies to block CD47 resulting in diminished tumor size in epithelial tumor models [32].

**Fig. 7:**
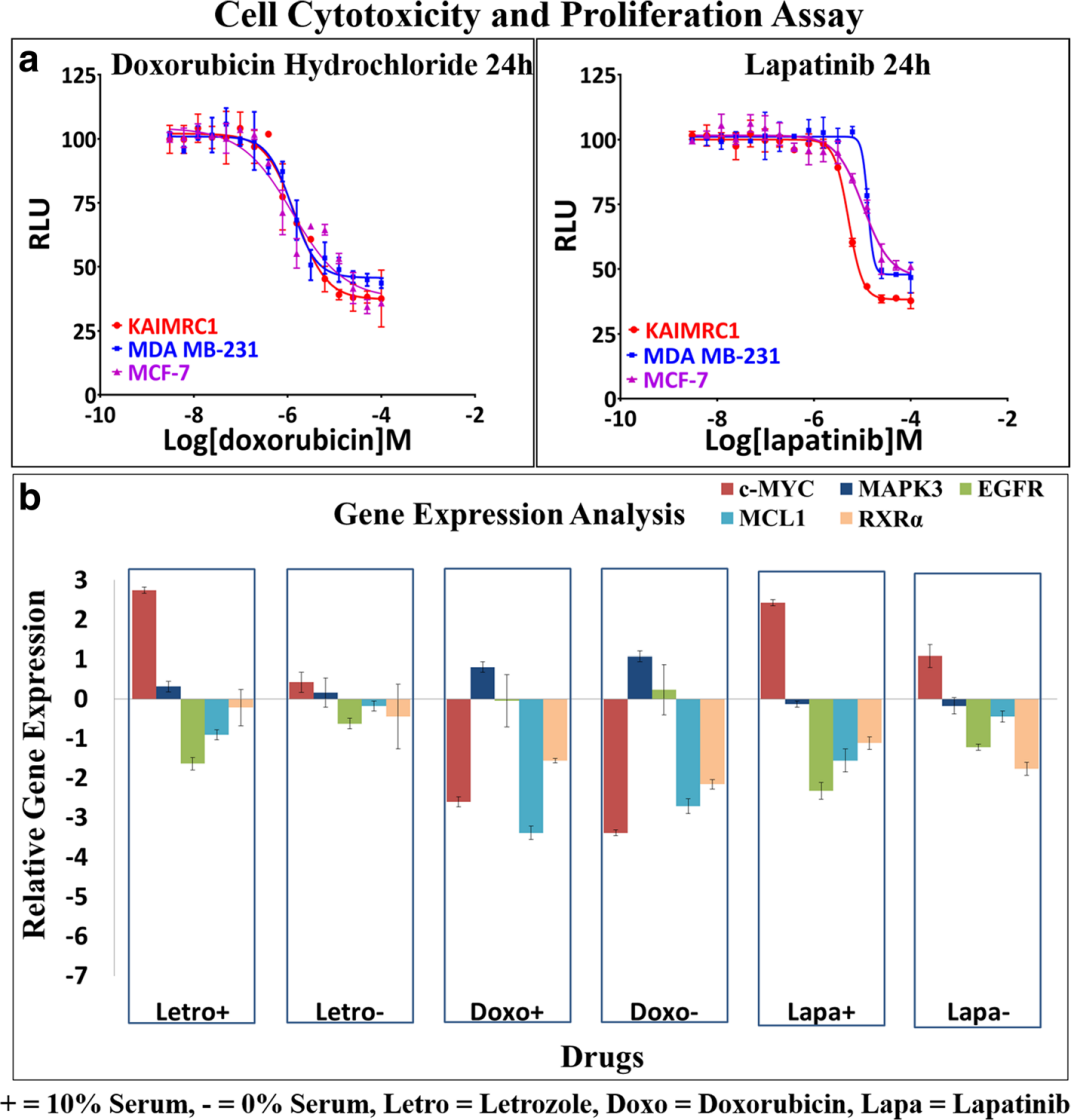
**a** Analysis of drug sensitivity of KAIMRC1 cells. Doxorubicin hydrochloride and Lapatinib were chosen to test drug sensitivity of KAIMRC1 cells in comparison to MDA MB-231 and MCF-7. MTT assay was performed to assess cytotoxicity and cell viability after drug treatment. Drug treatment was performed for 24 h prior to MTT assay in complete media. All three cell lines showed almost the same response to doxorubicin however they responded differently to lapatininb treatment. This result was later validated in breast cancer gene expression analysis. KAIMRC1 cells seems to be the most sensitive among the three cell lines with an IC_50_ of 5.175^e-006^ M. X-axis = Relative light unit (RLU) and y-axis = Logarithm of the drug molarity (Log[drug]M). **b** Gene expression analysis of human breast cells in response to drug treatment. Breast cancer cells KAIMRC-1 were treated with 10 μM of Letrozole, Doxorubicin hydrochloride and Lapatinib with and without serum in complete media. Cells were harvested after 6 h, RNA was isolated and cDNA was synthesized. Real time qPCR was performed using MAPKinase and AKT pathway associated genes to study gene expression changes in response to drug treatment. KAIMRC1 cells were found to be more responsive to drug treatment in comparison to other established cell lines. Treatment of KAIMRC1 cells with Lapatinib showed down regulation of EGFR, MCL1 and RXRα genes which explains the sensitivity of these cells to Lapatinib shown in (**b**). Letrozole and Doxorubicin showed no difference as compared to other cell lines. X-axis = Drugs and y-axis = Relative gene expression (ΔΔC_t_) to DMSO control. *n = 3* in all cases

Gene expression analysis after doxorubicin and letrozole treatment on KAIMRC1 cells showed no significant difference when compared to other breast cancer cell lines but treatment with lapatinib showed downregulation of MCL-1 and EGFR genes which correlates with the sensitivity shown by KAIMRC1 cells in the drug sensitivity assay (Fig. 7a). Lapatinib is a known inhibitor of EGFR which may explain the downregulation of the EGFR gene. On the other hand, downregulation of the MCL-1 gene was interesting as it results in activation of the apoptosis signaling pathway [33]. Whereas, increased expression of this gene results in high grade tumor and poor survival rate of breast cancer patients [34]. Recently it has been reported that breast cancer cells rely on MCL-1 for survival [35]. Another report suggested that inhibition of MCL-1 enhances lapatinib toxicity and overcomes lapatinib resistance [33].

We have identified 46 genes that were differentially expressed in KAIMRC1 cells. These genes were further divided into two groups based on their up- and down-regulation. Expression of ABCB1, ABCG2, AR, BIRC5, GLI1, MMP2, MMP9, RARB and SLIT2 was significantly high. It is noteworthy that most of these genes are associated with tumor suppression, cancer resistance, increased cancer cell growth and migration [36, 37]. Multidrug resistant (MDR) proteins, ABCB1 and ABCG2 are breast cancer resistance genes. The patient was on maintained on Letrozole and that may be the reason for the upregulation of MDR genes. It has been proposed that PI3K/AKT signaling may be critical in the functional regulation of MDR genes [38]. MMPs are associated with cancer cell invasion and metastasis [39].Whereas, slit homolog 2 (SLIT2) is a tumor suppressor gene [40]. Androgen receptor (AR) is present in almost 60–70% breast cancers [41] and can play a role as a marker for breast cancer along with ER and PR. Baculoviral inhibitor of apoptosis repeat-containing 5 (BIRC5) is associated with high proliferation levels and has been used as a prognosis marker lately [42]. Glioma-associated oncogene 1 (GLI1) is an oncogene and associated with CSCs [43]. Upregulation of this gene is associated with the epithelial to mesenchymal transition (EMT). The expression of RARB is usually low in breast cancers [44] but it was observed to be high in KAIMRC1 cell. The genomic instability and complexity of the cancer cells may be the result of the contradictory dysregulation of these genes that give rise to make the KAIMRC1 naturally transformed.

Interestingly, KAIMRC1 cells also showed increase in gene expression of BRCA1 and BRCA2, tumor suppressor genes. The upregulation of both the genes hints towards the initiation of the DNA damage repair mechanism of the cells. The functions of BRCA proteins are also linked to specific phosphorylation events although the extent to which phosphorylation-activated molecular pathways contribute to tumor suppression activity is not clear [45]. The constitutively active state of AKT in KAIMRC1 cells may be linked to tumor suppression activity of these cells.

On the other hand significant downregulation of KRT5 which is associated with relapse and reduced survival rate [46] suggests that these cells are less likely to be involved in promoting metastasis. Whereas, low expression of SFRP1, a tumor suppressor [47] explains the rapid proliferation characteristics of these cells.

Taken together, these findings and bioinformatics pathway analysis led to the conclusion that upregulation of certain genes in KAIMRC1 cells may be associated with DNA repair and particular signal transduction pathways. Conversely, downregulation of the above mentioned genes may be associated with cell cycle progression and disease related pathways.

In addition, we are currently developing KAIMRC1 mouse models to study if these cells are able to metastasize. Genome and exome analysis is also underway to identify unique genes associated with the natural transformation of these cells.

## Conclusions

In this study, we proposed for the first time a new naturally transformed cell line, KAIMRC1 of Saudi Arabian origin. A concise characterization of cell growth, clonogenicity, immunophenotyping, and karyotyping and drug sensitivity has demonstrated that the cell line carried distinct features. These cells showcased constitutively active state of AKT suggesting that AKT pathway may be one of the key player in their natural transformation into cancer cells. This cell line also expresses estrogen and progesterone receptors, and biomarkers, like CD44, CD47 and CD327 confirming their breast epithelial origin including CSCs marker ALDH1A1 expression. Another interesting feature of these cells is their growth ability in both monolayer and 3-D domes. Thus, KAIMRC1 provide a unique opportunity to study the development of early stage breast cancer particularly in the Arab population.

## Additional file

Additional file 1: Figure S1. Classification of KAIMRC1 cells. Western blot analysis of expression of estrogen receptor (ER), progesterone receptor (PR) and human epidermal growth factor receptor 2 (HER2) showed that KAIMRC1 cells are ER and PR positive and HER2 negative. This result validated our immunocytochemistry results. (TIFF 31 kb)

## Abbreviations

ABCB1: ATP Binding Cassette Subfamily B Member 1
ABCG2: ATP Binding Cassette Subfamily G Member 2
ALDH: Aldehyde Dehydrogenase
APC: Allophycocyanin
AR: Androgen receptor
ATCC: American type culture collection
Au/Pd: gold/palladium
BD: Becton Dickinson
BIRC5: Baculoviral inhibitor of apoptosis repeat-containing 5
CAFs: Cancer associated fibroblasts
CD: Cluster of differentiation
cDNA: Complimentary Deoxyribonucleic Acid
CK: Cytokeratin
cLSM: Confocal Laser Scanning Microscopy
CSCs: Cancer stem cells
ddH_2_O: Double distilled water
DMEM: Dulbecco’s Modified Eagle Medium
DMSO: Dimethyl Sulfoxide
ECM: Extracellular matrix
EGFR: Epidermal growth factor receptor
EMT: Epithelial to mesenchymal transition
ER: Estrogen receptor
ERK: Extracellular signal-regulated kinases
FACS: Fluorescence-activated cell sorting
FBS: Fetal Bovine Serum
FITC: Fluorescein isothiocyanate
FSC: Forward scatter
GLI1: Glioma-associated oncogene 1
GSK: Glycogen synthase kinase
HER2: Human epidermal growth factor receptor 2
IC_50_: Inhibitory concentration
ICC: Immunocytochemistry
IHC: Immunohistochemistry
IRB: Institutional Review Board
KAIMRC: King Abdullah International Medical Research Center
KRT5: Keratin 5
kV: Kilo volts
MAPK: Mitogen-activated protein kinases
MCF-7: Michigan Cancer Foundation-7
MCL-1: Myeloid cell leukemia sequence 1
MDR: Multidrug resistant
MMPs: Matrix metalloproteinases
PBS: Phosphate Buffered Saline
PCR: Polymerase chain reaction
PE: Phycoerythrin
PR: Progestrone receptor
RARB: Retinoic acid receptor beta
RNA: Ribonucleic acid
RQ: Relative quantification
SD: Standard deviation
SEM: Scanning Electron Microscopy
SFRP1: Secreted Frizzled Related Protein 1
SLIT2: Slit Guidance Ligand 2
SSC: Side scatter
SSEA: Stage-specific embryonic antigen-1
ΔΔCt: Delta Delta Threshold cycle

## References

[1] Kallioniemi A, Kallioniemi O, Sudar D, Rutovitz D, Gray J, Waldman F, Pinkel D (1992). Comparative genomic hybridization for molecular cytogenetic analysis of solid tumors. Science.

[2] Weiss MM, Hermsen MA, Meijer GA, van Grieken NC, Baak JP, Kuipers EJ, van Diest PJ (1999). Comparative genomic hybridisation. Mol Pathol.

[3] Makki J (2015). Diversity of breast carcinoma: histological subtypes and clinical relevance. Clinical medicine insights. Pathology.

[4] Lasfargues EY, Ozzello L (1958). Cultivation of human breast carcinomas. J Natl Cancer Inst.

[5] Smith HS, Wolman SR, Hackett AJ (1984). The biology of breast cancer at the cellular level. Biochim Biophys Acta.

[6] Smith HS, Wolman SR, Dairkee SH, Hancock MC, Lippman M, Leff A, Hackett AJ (1987). Immortalization in culture: occurrence at a late stage in the progression of breast cancer. J Natl Cancer Inst.

[7] Leibovitz A, Park J-G, Gazdar A (1994). 6 - cell lines from human breast A2 - hay, Robert J. Atlas of human tumor cell lines.

[8] Gazdar AF, Kurvari V, Virmani A, Gollahon L, Sakaguchi M, Westerfield M, Kodagoda D, Stasny V, Cunningham HT, Wistuba II (1998). Characterization of paired tumor and non-tumor cell lines established from patients with breast cancer. Int J Cancer.

[9] Mosoyan G, Nagi C, Marukian S, Teixeira A, Simonian A, Resnick-Silverman L, DiFeo A, Johnston D, Reynolds SR, Roses DF (2013). Multiple breast cancer cell-lines derived from a single tumor differ in their molecular characteristics and tumorigenic potential. PLoS One.

[10] Pandrangi SL, Raju Bagadi SA, Sinha NK, Kumar M, Dada R, Lakhanpal M, Soni A, Malvia S, Simon S, Chintamani C (2014). Establishment and characterization of two primary breast cancer cell lines from young Indian breast cancer patients: mutation analysis. Cancer Cell Int.

[11] Shen C, Gu M, Liang D, Miao L, Hu L, Zheng C, Chen J (2009). Establishment and characterization of three new human breast cancer cell lines derived from Chinese breast cancer tissues. Cancer Cell Int.

[12] Cox BD, Natarajan M, Stettner MR, Gladson CL (2006). New concepts regarding focal adhesion kinase promotion of cell migration and proliferation. J Cell Biochem.

[13] Marcato P, Dean CA, Pan D, Araslanova R, Gillis M, Joshi M, Helyer L, Pan L, Leidal A, Gujar S (2011). Aldehyde dehydrogenase activity of breast cancer stem cells is primarily due to isoform ALDH1A3 and its expression is predictive of metastasis. Stem Cells.

[14] Yan Y, Zuo X, Wei D (2015). Concise review: emerging role of CD44 in cancer stem cells: a promising biomarker and therapeutic target. Stem Cells Transl Med.

[15] Ruvolo PP, Qiu Y, Coombes KR, Zhang N, Neeley ES, Ruvolo VR, Hail N, Borthakur G, Konopleva M, Andreeff M (2015). Phosphorylation of GSK3α/β correlates with activation of AKT and is prognostic for poor overall survival in acute myeloid leukemia patients. BBA Clinical.

[16] Neve RM, Chin K, Fridlyand J, Yeh J, Baehner FL, Fevr T, Clark L, Bayani N, Coppe JP, Tong F (2006). A collection of breast cancer cell lines for the study of functionally distinct cancer subtypes. Cancer Cell.

[17] Ingber DE (2008). Can cancer be reversed by engineering the tumor microenvironment?. Semin Cancer Biol.

[18] Dontu G, Wicha MS (2005). Survival of mammary stem cells in suspension culture: implications for stem cell biology and Neoplasia. J Mammary Gland Biol Neoplasia.

[19] Keese CR, Wegener J, Walker SR, Giaever I (2004). Electrical wound-healing assay for cells in vitro. Proc Natl Acad Sci U S A.

[20] Lv Z, Bian Z, Shi L, Niu S, Ha B, Tremblay A, Li L, Zhang X, Paluszynski J, Liu M (2015). Loss of cell surface CD47 ‘clustering’ formation and binding avidity to SIRPα facilitate apoptotic cell clearance by macrophage. J Immunol (Baltimore, Md : 1950).

[21] Singhai R, Patil VW, Jaiswal SR, Patil SD, Tayade MB, Patil AV (2011). E-Cadherin as a diagnostic biomarker in breast cancer. N Am J Med Sci.

[22] Basakran NS (2015). CD44 as a potential diagnostic tumor marker. Saudi Medical Journal.

[23] Al-Hajj M, Wicha MS, Benito-Hernandez A, Morrison SJ, Clarke MF (2003). Prospective identification of tumorigenic breast cancer cells. Proc Natl Acad Sci U S A.

[24] de Beca FF, Caetano P, Gerhard R, Alvarenga CA, Gomes M, Paredes J, Schmitt F (2013). Cancer stem cells markers CD44, CD24 and ALDH1 in breast cancer special histological types. J Clin Pathol.

[25] Olsson E, Honeth G, Bendahl P-O, Saal LH, Gruvberger-Saal S, Ringnér M, Vallon-Christersson J, Jönsson G, Holm K, Lövgren K (2011). CD44 isoforms are heterogeneously expressed in breast cancer and correlate with tumor subtypes and cancer stem cell markers. BMC Cancer.

[26] Ginestier C, Hur MH, Charafe-Jauffret E, et al. ALDH1 is a marker of normal and malignant human mammary stem cells and a predictor of poor clinical outcome. Cell stem cell. 2007;1(5):555–67. doi:10.1016/j.stem.2007.08.014. 10.1016/j.stem.2007.08.014 PMC2423808 18371393

[27] Zhao H, Wang J, Kong X, Li E, Liu Y, Du X, Kang Z, Tang Y, Kuang Y, Yang Z (2016). CD47 promotes tumor invasion and metastasis in non-small cell lung cancer. Sci Rep.

[28] Chao MP, Alizadeh AA, Tang C, Jan M, Weissman-Tsukamoto R, Zhao F, Park CY, Weissman IL, Majeti R (2011). Therapeutic antibody targeting of CD47 eliminates human acute lymphoblastic leukemia. Cancer Res.

[29] Steinert G, Schölch S, Niemietz T, Iwata N, García SA, Behrens B, Voigt A, Kloor M, Benner A, Bork U (2014). Immune escape and survival mechanisms in circulating tumor cells of colorectal cancer. Cancer Res.

[30] Lee TK-W, Cheung VC-H, Lu P, Lau EYT, Ma S, Tang KH, Tong M, Lo J, Ng IOL (2014). Blockade of CD47-mediated cathepsin S/protease-activated receptor 2 signaling provides a therapeutic target for hepatocellular carcinoma. Hepatology.

[31] Zhang H, Lu H, Xiang L, Bullen JW, Zhang C, Samanta D, Gilkes DM, He J, Semenza GL (2015). HIF-1 regulates CD47 expression in breast cancer cells to promote evasion of phagocytosis and maintenance of cancer stem cells. Proc Natl Acad Sci.

[32] Edris B, Weiskopf K, Volkmer AK, Volkmer JP, Willingham SB, Contreras-Trujillo H, Liu J, Majeti R, West RB, Fletcher JA (2012). Antibody therapy targeting the CD47 protein is effective in a model of aggressive metastatic leiomyosarcoma. Proc Natl Acad Sci U S A.

[33] Cerella C, Muller F, Gaigneaux A, Radogna F, Viry E, Chateauvieux S, Dicato M, Diederich M (2015). Early downregulation of Mcl-1 regulates apoptosis triggered by cardiac glycoside UNBS1450. Cell Death Dis.

[34] Ding Q, He X, Xia W, Hsu J-M, Chen C-T, Li L-Y, Lee D-F, Yang J-Y, Xie X, Liu J-C (2007). Myeloid cell Leukemia-1 inversely correlates with glycogen Synthase Kinase-3β activity and associates with poor prognosis in human breast cancer. Cancer Res.

[35] Xiao Y, Nimmer P, Sheppard GS, Bruncko M, Hessler P, Lu X, Roberts-Rapp L, Pappano WN, Elmore SW, Souers AJ (2015). MCL-1 is a key determinant of breast cancer cell survival: validation of MCL-1 dependency utilizing a highly selective small molecule inhibitor. Mol Cancer Ther.

[36] Egeblad M, Werb Z (2002). New functions for the matrix metalloproteinases in cancer progression. Nat Rev Cancer.

[37] Lee JY, Park K, Lee E, Ahn T, Jung HH, Lim SH, Hong M, Do I-G, Cho EY, Kim D-H (2016). Gene expression profiling of breast cancer brain metastasis. Sci Rep.

[38] Nakanishi T, Ross DD (2012). Breast cancer resistance protein (BCRP/ABCG2): its role in multidrug resistance and regulation of its gene expression. Chin J Cancer.

[39] Freije JM, Balbin M, Pendas AM, Sanchez LM, Puente XS, Lopez-Otin C (2003). Matrix metalloproteinases and tumor progression. Adv Exp Med Biol.

[40] Yu J, Cao Q, Yu J, Wu L, Dallol A, Li J, Chen G, Grasso C, Cao X, Lonigro RJ (2010). The neuronal repellent SLIT2 is a target for repression by EZH2 in prostate cancer. Oncogene.

[41] Iacopetta D, Rechoum Y, Fuqua SAW (2012). The role of androgen receptor in breast cancer. Drug discovery today. Dis Mech.

[42] Ghaffari K, Hashemi M, Ebrahimi E, Shirkoohi R (2016). BIRC5 genomic copy number variation in early-onset breast cancer. Iran Biomed J.

[43] Colavito SA, Zou MR, Yan Q, Nguyen DX, Stern DF (2014). Significance of glioma-associated oncogene homolog 1 (GLI1)expression in claudin-low breast cancer and crosstalk with the nuclear factor kappa-light-chain-enhancer of activated B cells (NFκB) pathway. Breast Cancer Res.

[44] Widschwendter M, Berger J, Muller HM, Zeimet AG, Marth C (2001). Epigenetic downregulation of the retinoic acid receptor-beta2 gene in breast cancer. J Mammary Gland Biol Neoplasia.

[45] Yoshida K, Miki Y (2004). Role of BRCA1 and BRCA2 as regulators of DNA repair, transcription, and cell cycle in response to DNA damage. Cancer Sci.

[46] Alshareeda AT, Soria D, Garibaldi JM, Rakha E, Nolan C, Ellis IO, Green AR (2013). Characteristics of basal cytokeratin expression in breast cancer. Breast Cancer Res Treat.

[47] Matsuda Y, Schlange T, Oakeley EJ, Boulay A, Hynes NE (2009). WNT signaling enhances breast cancer cell motility and blockade of the WNT pathway by sFRP1 suppresses MDA-MB-231 xenograft growth. Breast Cancer Res.

