# Supplementary figures and images for: Isolation and characterization of a new naturally immortalized human breast carcinoma cell line, KAIMRC1
Source: BMC Cancer. 2017 Nov 29;17:803. doi: 10.1186/s12885-017-3812-5 (PMC5707794; doi:10.1186/s12885-017-3812-5)

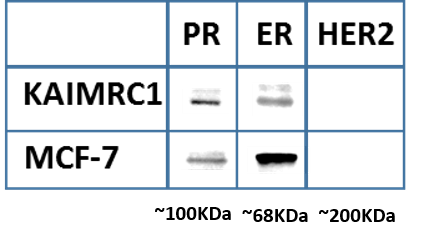

Supplement: Additional file 1: Figure S1. — Classification of KAIMRC1 cells. Western blot analysis of expression of estrogen receptor (ER), progesterone receptor (PR) and human epidermal growth factor receptor 2 (HER2) showed that KAIMRC1 cells are ER and PR positive and HER2 negative. This result validated our immunocytochemistry results. (TIFF 31 kb) [file 12885_2017_3812_MOESM1_ESM.tif]
